# Phenotypic characterization and whole genome analysis of extended-spectrum beta-lactamase-producing bacteria isolated from dogs in Germany

**DOI:** 10.1371/journal.pone.0206252

**Published:** 2018-10-26

**Authors:** Tim Boehmer, Amy J. Vogler, Astrid Thomas, Sabine Sauer, Markus Hergenroether, Reinhard K. Straubinger, Dawn Birdsell, Paul Keim, Jason W. Sahl, Charles H. D. Williamson, Julia M. Riehm

**Affiliations:** 1 Central Institute of the Bundeswehr Medical Service Munich, Garching, Bavaria, Germany; 2 The Pathogen and Microbiome Institute, Northern Arizona University, Flagstaff, Arizona, United States of America; 3 Institute of Infectious Diseases and Zoonoses, Department of Veterinary Sciences, Faculty of Veterinary Medicine, Ludwig-Maximilian University, Munich, Germany; 4 Bundeswehr Headquarter, Munich, Germany; St Petersburg Pasteur Institute, RUSSIAN FEDERATION

## Abstract

Asymptomatic colonization with extended-spectrum beta-lactamase (ESBL) producing Enterobacteriaceae has been described for humans, various mammal species, and birds. Here, antimicrobial resistant bacteria were recovered from dog feces originating in Germany, Kosovo, Afghanistan, Croatia, and Ukraine, with a subset of mostly *E*. *coli* isolates obtained from a longitudinal collection over twelve months. *In vitro* antimicrobial resistance testing revealed various patterns of resistance against single or all investigated beta-lactam antibiotics, with none of the 101 isolates resistant against two tested carbapenem antibiotics. Whole genome sequence analysis revealed bacteria species-specific patterns for 23 antimicrobial resistance coding DNA sequences (CDS) that were unapparent from the *in vitro* analysis alone. Phylogenetic analysis of single nucleotide polymorphisms (SNP) revealed clonal bacterial isolates originating from different dogs, suggesting transmission between dogs in the same community. However, individual resistant *E*. *coli* clones were not detected over a period longer than seven days. Multi locus sequence typing (MLST) of 85 *E*. *coli* isolates revealed 31 different sequence types (ST) with an accumulation of ST744 (n = 9), ST10 (n = 8), and ST648 (n = 6), although the world-wide hospital-associated CTX-M beta-lactamase producing ST131 was not detected. Neither the antimicrobial resistance CDSs patterns nor the phylogenetic analysis revealed an epidemiological correlation among the longitudinal isolates collected from a period longer than seven days. No genetic linkage could be associated with the geographic origin of isolates. In conclusion, healthy dogs frequently carry ESBL-producing bacteria, independent to prior treatment, which may be transmitted between individual dogs of the same community. Otherwise, these antimicrobial resistant bacteria share few commonalities, making their presence eerily unpredictable.

## Introduction

Beta-lactams are among the most popular antibiotics, worldwide, for the treatment of bacterial infections [1]. Unfortunately, multidrug-resistant bacteria producing extended-spectrum beta-lactamases (ESBL) are also prevalent worldwide [2]. Descriptions of ESBL isolates originating from patients in intensive care units of European hospitals were first published in the mid-1980s [1]. Since then, ESBL-producing Enterobacteriaceae have been identified from a plethora of sources, including humans, animals, food, feed, and other environmental sources [3–7].

ESBL-producing *Escherichia coli* isolated from dogs were first described in 1988, following treatment of the dogs with beta-lactam antibiotics [8]. Since then, the presence of ESBL-producing bacteria has been described repeatedly for sick, but also completely healthy companion animals, including dogs [4,9–13]. One longitudinal study occurring over six months identified a variety of ESBL-producing Enterobacteriaceae in healthy dogs with highly dynamic fecal shedding patterns, occurring either continuously or periodically [14].

Comprehensive characterization of ESBL-producing Enterobacteriaceae is critical for understanding transmission routes and persistence in potential reservoirs, as well as their potential to transfer multidrug-resistant genetic coding elements and/or cause disease [15]. To date, a variety of methods, including biochemistry, phage typing, serotyping, bacteriocin typing, analytical isoelectric focusing, and pulsed-field gel electrophoresis have been used for characterization [1, 9, 16–17]. However, the discriminatory power of these methods has been incomplete and the reproducibility among different laboratories low, limiting insight into the epidemiology of these bacteria [1]. High throughput whole genome sequencing provides an opportunity to gather much more comprehensive data on antimicrobial resistance carrying genetic elements in various bacteria. And, when coupled with appropriate epidemiological data, should allow for greater insight into the population dynamics of ESBL-producing bacteria [17–19]. In this study, we combine *in vitro* diagnostics with whole genome analysis to investigate the genetic diversity and antimicrobial resistance profiles of ESBL-producing bacteria from dogs living in close proximity to humans and gain a greater understanding of this overlooked source of antimicrobial resistance.

## Material and methods

### Strain isolation

ESBL-producing bacteria were exclusively isolated from fresh canine feces. As the dogs were not at all touched for this purpose, the Institutional Animal Care and Use Committee (IACUC) was not involved. The authorization of the sample collection regarding animals within any North Atlantic Treaty Organization (NATO) theatre of operations was given by direct NATO order and was to be executed by the military veterinary authorities, here authors of the present study, that must review which diseases were prevalent in the area to which animals will be deployed [20]. The collection within Germany was carried out within the area of caserns or on private land in the presence of and in accordance to the commanding officer or the respective landlord.

Dog feces from a community of 17 German (GER) military dogs, and from three additional military dogs living in a different community, was sampled over a twelve-month period, from April 2015 to March 2016. Within this longitudinal subset, fecal samples of all dogs were screened daily within the first week of investigation, then weekly during the first month, then monthly for six months, and, finally, once at the end of twelve months. The sampled dogs had no history of treatment over the previous twelve months. Additional, sporadic samples were collected from military dogs from other locations, including Croatia (CRO) and Ukraine (UKR), and also from stray dogs from military operation zones in Afghanistan (AFG) and Kosovo (KOS) (S1 Table). Samples were collected from dog feces directly after voiding, and were processed in the laboratory within a maximum of six hours. Initial screening of fecal samples was carried out by direct inoculation on a selective Brilliance ESBL AGAR (Oxoid, Wesel, Germany) containing an antibiotic-mix, using a 10 μl inoculation loop. Plates were incubated at 37°C, and putative isolates were harvested based on their colony morphology after 24 h according to the manufacturer’s instructions. All morphologically suspicious isolates were picked, with at least three morphologically indistinguishable isolates selected per plate, if available. Selected isolates were then sub-cultured on Columbia sheep blood agar (Oxoid, Wesel, Germany). The tentative species of each isolate was determined via mass spectrometry using a MALDI Biotyper system (Bruker, Bremen, Germany).

The isolates were named according to their geographic origin (GER, UKR, KOS, CRO, AFG), individual source (military dog—MD, stray dog–SD, stray fox–SF, companion dog–CD, environmental–EN, and number indicating specific animal), year and month of isolation, bacterial species and a running number within the present project; e.g. GER_MD06_1505_Eco_007 (S1 Table).

Whole genome analysis confirmed the species identification for the investigated isolates except for two isolates: the *Enterobacter kobei* isolate GER_MD16_1505_Esp_090 was labeled *Enterobacter* sp. and the *Pseudomonas fulva* isolate AFG_SD02_1510_Psp_092 was labeled *Pseudomonas* sp. due to low measures of identity to reference genomes for each species.

### *In vitro* antimicrobial susceptibility testing

All recovered isolates were tested *in vitro* for their antimicrobial resistance profile using the commercially available standard micro-dilution system, MICRONAUT-S Beta-Lactamases (Merlin, Berlin, Germany). This method included tests for six different singular antimicrobial substances, including, cefoxitin (COX), cefotaxime (CTX), ceftazidime (CAZ), cefepime (CEP), ertapenem (ERT), and meropenem (MER), and three additional combinations comprised of CTX, CAZ, and CEP tested in combination with clavulanic acid. The minimum inhibitory concentration (MIC) was determined for each isolate in accordance with the manufacturer’s directions (Merlin, Berlin, Germany).

To assess the presence of multiple beta-lactamases (multiple resistance determinants) MIC values were interpreted according to the breakpoint-value standards set for drug selection and interpretation by the Clinical and Laboratory Standards Institute (CLSI, Wayne, PA, USA). Specifically, the CLSI *Methods for Dilution Antimicrobial Susceptibility Tests for Bacteria that Grow Aerobically*, 27^th^ Informational Supplement (M100-S27), and the VET01/ VET01-S2 guidelines, *Performance Standards for Antimicrobial Disk and Dilution Susceptibility Tests for Bacteria Isolated From Animals*, presented by the Subcommittee on Veterinary Antimicrobial Susceptibility Testing were used [21–22]. Complete breakpoint values were solely available for *E*. coli and P. mirabilis and partially available for CEP and CAZ for *Pseudomonas aeruginosa*. However, no breakpoints were available for *Enterobacter* spp., *Aeromonas* sp. and *Pseudomonas* sp. other than *P*. *aeruginosa* [21]. By definition, extended-spectrum beta-lactamase is produced by a bacterium if more than a three twofold concentration decrease in a MIC is observed for either antimicrobial agent tested in combination with clavulanic acid versus the MIC of the agent when tested alone [22].

### Genome sequencing, assembly, MLST

Whole genome sequencing was attempted for all isolates. DNA was extracted using the QIAamp DNA Mini Kit (QIAGEN, Hilden, Germany), and sequenced on the Illumina MiSeq and NextSeq platforms (TGen, Flagstaff, AZ, USA). Raw sequence data were assembled with a pipeline that includes Trimmomatic for read trimming [23], SPAdes v3.10.1 for contig assembly [24], Pilon for assembly polishing [25], and BLAST to identify potential sequence contamination [26]. *In silico* multi-locus sequencing typing (MLST) of identified *E*. *coli* genomes was carried out using a custom script (https://gist.github.com/jasonsahl/2eedc0ea93f90097890879e56b0c3fa3) that utilizes BLAST and the PubMLST database (https://pubmlst.org/) for *Escherichia coli* [26–27].

### Screening for CDS associated with antimicrobial resistance and virulence factors

All recovered genome assemblies were screened for antibiotic resistance genes with ABRicate (https://github.com/tseemann/abricate), using the ResFinder database (downloaded 2017 July 8) [28]. In addition, virulence gene profiles were determined by screening the genome assemblies for selected virulence coding DNA sequences (CDSs) with the large-scale blast score ratio (LS-BSR) pipeline [29] using the BLAT alignment option [30]. Screened virulence genes included 35 publicly available CDSs for fimbriae, toxins, and other proteins responsible for adhesion, agglutination, gene transfer, or iron acquisition. A CDS was considered as present within a genome if the blast score ratio was above 0.8 [31].

### Phylogenetic analyses

Phylogenetic analyses were applied to all of the recovered *E*. *coli* sequences to identify genetic relationships among the isolates. The *E*. *coli* genomes were compared to a reference, K-12 W3110 (GCA_000010245.1), and core genome single nucleotide polymorphisms (SNPs) were identified [32]. Specifically, sequencing reads were aligned to the reference with BWA-MEM [33]. SNPs were called using the UnifiedGenotyper method in GATK [34–35]. Putative SNP positions with less than 10X coverage or allele proportions less than 90% were filtered from the analysis. Any SNP identified from duplicated regions of the reference, as identified through NUCmer [36] self-alignments, were filtered from downstream analyses. All of the SNP detection methods were performed in conjunction with the NASP pipeline [37]. Phylogenies were inferred from the identified SNPs with IQ-TREE v 1.4.4 using the identified best-fit model, TVM+ASC+G4 (S3 Table) [38].

## Results

### Isolates

In total, 101 bacterial isolates were recovered using the selective Brilliance ESBL AGAR between January 2015 and June 2016 (S1 Table). Of these, 75 originated from 16 German military dogs, with an additional five originating from two companion dogs (GER_CD71, GER_CD72) living in the same household as German military dog GER_MD77. Of the foreign isolates, six originated from stray dogs (n = 2), shelter dogs (n = 3), and a stray fox (n = 1) in Kosovo; eight originated from stray dogs in Afghanistan; three originated from a Ukrainian military dog located in Kosovo; and two originated from a Croatian military dog located in Afghanistan (S1 Table). Two additional isolates originated from routine hygiene samples in Germany, and were considered as outgroups of non-animal origin.

Isolates were recovered from 16 of the 20 tested German military dogs. However, repeat isolation of ESBL-producing bacteria from samples taken on different dates was only successful for five of the 17 German military dogs in the longitudinal study (29%) (S1 Table). The longitudinal collection identified twelve isolates from GER_MD01 over a period of eleven months, five isolates from GER_MD02 over a period of seven months, ten isolates from GER_MD03 over a period of three months, four isolates from GER_MD06 over a period of seven months, and eight isolates from GER_MD14 over a period of seven months (S1 Table).

A total of 31 isolates were collected from either the same dog or household within a single month of sampling, allowing for an examination of ESBL-diversity within a single dog and/or household over a short period of time. These included three isolates from GER_MD07, three isolates from GER_MD08, 13 isolates from GER_MD11, two isolates from GER_MD17, seven isolates from GER_MD77 or his companions GER_CD71 and GER_CD72, and three isolates from UKR_MD01 (S1 Table).

Identification of the 101 isolates revealed 93 *Escherichia coli*, one *Proteus mirabilis*, two *Enterobacter cloacae*, one *Enterobacter* sp. (all family Enterobacteriaceae), one *Aeromonas caviae*, one *Aeromonas hydrophila*, one *Pseudomonas aeruginosa*, and one *Pseudomonas* sp. (S1 Table). For two isolates, the identification was possible only on genus level due to contradictory results based on the MALDI-TOF and the molecular approach.

### Antimicrobial susceptibility

MICs for the entire antibiotic test panel were recovered for all isolates (S2 Table). Interpretation of MICs was carried out for the 94 isolates (*E*. *coli*, *P*. *mirabilis*) in accordance with CLSI criteria. Due to limited or missing MIC values in the CLSI guidelines, interpretation was restricted for CAZ and CEP for the *P*. *aeruginosa* isolate and could not be performed for the *Enterobacter*, *Aeromonas* and *Pseudomonas* non-*aeruginosa* isolates (S2 Table). For COX, representing cephamycin antibiotics within the 2^nd^ generation of cephalosporins, 13 isolates (14%) were resistant and 81 (86%) were susceptible (S2 Table). For CTX, representing 3^rd^ generation cephalosporins, 91 isolates (97%) were resistant, one (1%) was susceptible, and two (2%) had an intermediate state. For CAZ, 34 isolates (36%) were resistant, 51 (54%) were susceptible, and ten had an intermediate state (S2 Table). For CEP, a 4^th^ generation cephalosporin, 87 isolates (92%) were resistant, six (6%) were susceptible, and two (2%) had an intermediate state (S2 Table). For the carbapenems, ERT and MER, all tested isolates were susceptible (S2 Table). Tests of CTX, CAZ, and CEP with the addition of 4 μg/ml clavulanic acid to inhibit beta-lactamase activity, revealed 88 (93%) out of 95 isolates to be real ESBL-producers in the *in vitro* system and according to the CLSI guidelines (S2 Table) [21–22]. One isolate, GER_MD01_1509_Eco_059, was susceptible to all of the tested substances (S2 Table).

### Genome assembly and CDS identification

Draft genome assemblies were generated for 93 isolates, with the remaining eight isolates excluded due to poor sequence quality (S3 Table). Of these, 85 were identified as *E*. *coli* genomes. Genome assemblies were submitted to GenBank and raw data was submitted to the sequence read archive (see S3 Table for individual accession numbers).

Use of ABRicate and the ResFinder database revealed sequence hits for 23 of 1,309 screened CDSs for beta-lactamases amongst the genome assemblies (Table 1 and S4 Table). Regarding class A beta-lactamase genes, 74 of 85 analyzed *E*. *coli* genomes possessed at least one CTX-M-type beta-lactamase CDS, with 33 positive for *bla*_CTX-M-1_, 28 positive for *bla*_CTX-M-15_, eleven positive for *bla*_CTX-M-14_, two positive for *bla*_CTX-M-3_, and one positive for *bla*_CTX-M-2_. One isolate was positive for *bla*_SFO-1_ and another for *bla*_SHV-12_. Forty isolates possessed *bla*_TEM-1_-type beta-lactamase CDSs, with two positive for *bla*_TEM-1A_ and 38 positive for *bla*_TEM-1B_ (Table 1 and S4 Table). Regarding class B beta-lactamases, one *A*. *hydrophilia* genome was positive for *bla*_Cph-A1_ and three *E*. *coli* genomes were positive for *bla*_VIM-1_ (Table 1). Ten genomes were positive for class C beta-lactamase CDSs, including three *E*. *coli* genomes positive for *bla*_ACC-1_ and single isolates positive for *bla*_ACT-7_, *bla*_ACT-14_, *bla*_CMY-2_, *bla*_MIR-6_, *bla*_MOX-5_, *bla*_MOX-6_, *bla*_PAO_, and *ampH*, respectively (Table 1 and S4 Table). Twenty isolates were positive for class D beta-lactamase CDSs, including 18 *E*. *coli* genomes positive for *bla*_OXA-1_, one *P*. *aeruginosa* positive for *bla*_OXA-50_, and one *A*. *caviae* positive for *bla*_OXA-504_ (Table 1 and S4 Table). Only two isolates, the GER_MD10_1505_Pmi_049, and the GER_EN02_1501_Eco_088 were negative for all of the 1,309 screened beta-lactamase CDSs (Table 1 and S4 Table).

**Table 1:** Prevalence of 23 specific beta-lactamase (BL) genes coding for antimicrobial resistance showing a clear species specificity (also S4 Table).

category	BL, extended-spectrum BL (ESBL)	specific resistance gene	positive strains (n) out of 93	bacterial species
class A BL (penicillinase)	cefotaximase-Munich, ESBL	*bla*_CTX-M-1_	33	*Escherichia coli*
		*bla*_CTX-M-2_	1	*Escherichia coli*
		*bla*_CTX-M-3_	2	*Escherichia coli*
		*bla*_CTX-M-14_	11	*Escherichia coli*
		*bla*_CTX-M-15_	28	*Escherichia coli*
	*Serratia fonticola* class A BL	*bla*_SFO-1_	1	*Aeromonas hydrophila*
	sulphydryl variable class A BL	*bla*_SHV-12_	1	*Escherichia coli*
	Temoneira, class A BL	*bla*_TEM-1A_	2	*Escherichia coli*
		*bla*_TEM-1B_	38	*Escherichia coli*
class B BL (carbapenemase)	carbapenem-hydrolyzing metallo BL	*bla*_Cph-A1_	1	*Aeromonas hydrophila*
zinc dependent	Verona integron-encoded metallo BL	*bla*_VIM-1_	3	*Escherichia coli*
class C BL (cephalosporinase)	Ambler class C-1 cephalosporin-hydrolyzing class C BL	*bla*_Acc-1_	3	*Escherichia coli*
	ampicillin type cephalosporin-hydrolyzing class C BL	*bla*_Act-7_	1	*Enterobacter cloacae*
		*bla*_Act-14_	1	*Enterobacter cloacae*
	aminopenicillin-inactivating (Amp) cephalosporinase	*bla*_AmpH_	1	*Aeromonas hydrophila*
	cephamycinase, plasmid derived pYMG-1 *bla*	*bla*_CMY-2_	1	*Escherichia coli*
	methoxy-/ imino-Res; cephalosporin-hydrolyzing class C BL	*bla*_MIR-6_	1	*Enterobacter* sp.
	moxalactam-inactivating cephalosporinase	*bla*_MOX-5_	1	*Aeromonas hydrophila*
		*bla*_MOX-6_	1	*Aeromonas caviae*
	*Pseudomonas aeruginosa* cephalosporinase	*bla*_PAO_	1	*Pseudomonas aeruginosa*
class D BL	oxacillin-hydrolyzing BLs	*bla*_OXA-1_	18	*Escherichia coli*
		*bla*_OXA-50_	1	*Pseudomonas aeruginosa*
		*bla*_OXA-504_	1	*Aeromonas caviae*

Patterns in the identified antimicrobial resistance CDSs suggested bacterial species specificity (Table 1). The two *E*. *cloacae* isolates were the only isolates to possess *bla*_ACT-7_ and *bla*_ACT-14_, respectively. Likewise, the further *Enterobacter* sp. isolate was the only isolate to possess *bla*_MIR-6_, the *P*. *aeruginosa* isolate was the only isolate to possess *bla*_PAO_ and *bla*_OXA-50_, and the *A*. *caviae* isolate was the only isolate positive for *bla*_MOX-6_ and *bla*_OXA-504_. Similarly, the *A*. *hydrophila* isolate was the only isolate positive for *bla*_SFO-1_, *bla*_Cph-A1_, *bla*_MOX-5_, and *ampH*, four antimicrobial resistance coding beta-lactamase genes and the highest number detected in a single isolate (Table 1).

Sequence hits for seven of 35 screened virulence CDSs were detected among the genome assemblies originating from the present study group (Table 2). Sequence hits included eleven isolates positive for an adhesion protein CDS (i.e., the long polar fimbriae *lpfA*), one isolate positive for the agglutination protein temperature sensitive hemagglutinin CDS (*tsh*). A CDS catalyzing site specific integration into chromosome and responsible for horizontal gene transfer (*argW* tRNA gene) was detected in 20 of the isolates [39–40]. The CDS for iron acquisition, and iron carrier system, siderophore receptor A (*ireA*) was detected in four of the isolates [41]. Regarding toxin production, one isolate was positive for *E*. *coli* heat-stable (*ST*) enterotoxin A (*estA*) and the ETEC heat-stable enterotoxin (*STp*). Finally, four isolates were positive for *Shigella* enterotoxin B (*senB*) (Table 2). No isolate possessed more than two of the detected virulence CDSs (S1 Table).

**Table 2:** Virulence genes detected in the isolates of the present study using whole genome sequence analysis.

Protein function	Gene	Full name and effect	Number of isolates carrying the respective gene	Accession Number
adhesion	lpfA	long polar fimbriae	11	AB161111.1
agglutination	tsh	temperature sensitive hemagglutinin, autotransporter protein	1	AF218073.1
gene transfer	argW	tRNA gene; site specific integration into chromosome and horizontal gene transfer	20	U11296.1
iron acquisition	ireA	Siderophore, iron carrier receptor	4	KU295572.1
toxin	estA	*E*. *coli* heat stable toxin A/ +variant	1 (environment)	AF005091.1
toxin	STp	ETEC heat-stable enterotoxin	1 (environment)	FN649417.1:c57269-57051
toxin	senB	*Shigella* enterotoxin B	4	Z54195.1

### MLST, SNPs and phylogenetic analysis

**Fig 1:**
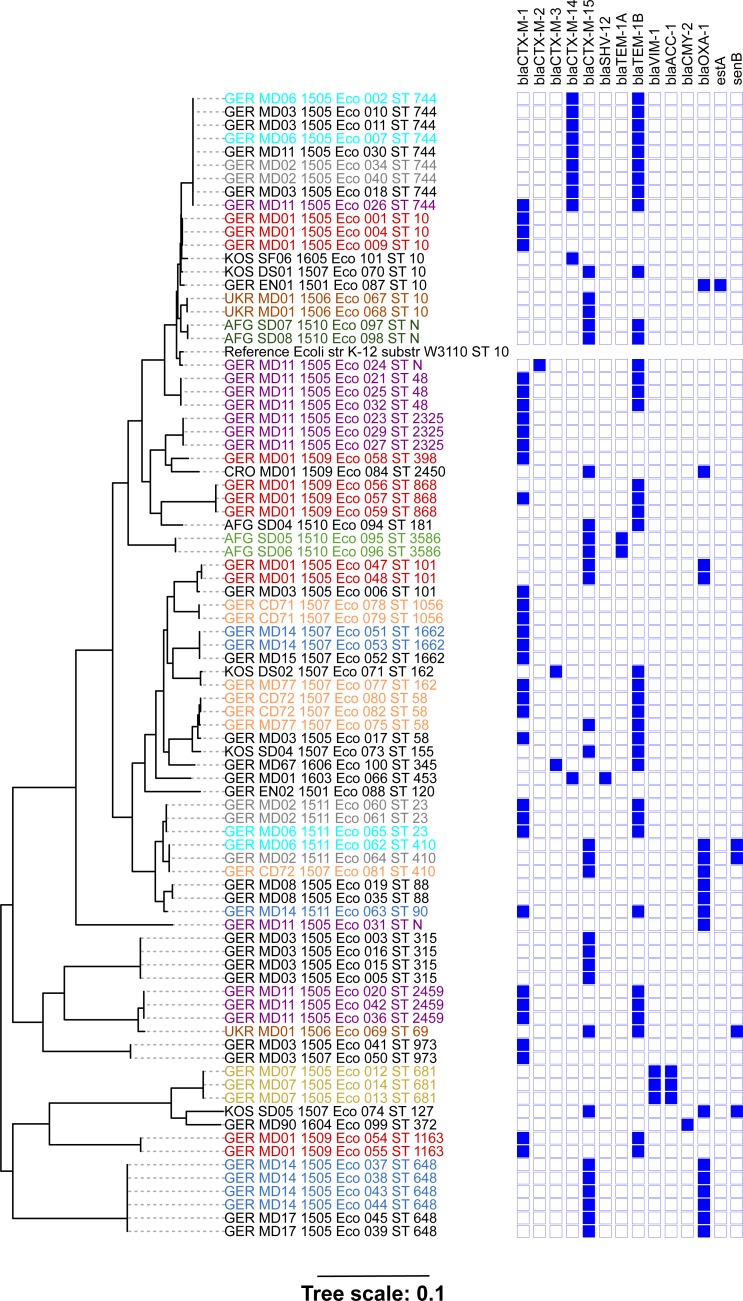
Phylogenetic tree of 85 ESBL-producing *E*. *coli*. Phylogenetic analysis based on 215,629 concatenated SNPs revealed clustering according to their MLST sequence type. *E*. *coli* K-12 substr W3110 was used as reference strain. All genome sequences except for the reference strain, and the environmental isolate GER_EN02_1501 revealed genes coding for antimicrobial resistance.

The MLST, SNP and phylogenetic analyses were limited to the *E*. *coli* sequences. Of the 85 identified *E*. *coli*genomes, 81 could be classified as one of 31 out of > 7,000 known *E*. *coli* sequence types (STs) based on MLST. The most frequently identified STs were ST744 (n = 9), ST10 (n = 8), ST648 (n = 6), ST58 (n = 4), and ST315 (n = 4), with the remaining 26 STs represented ≤3 times among the 85 genomes (Fig 1 and S1 Table). The remaining four *E*. *coli* genomes each contained one or two novel MLST alleles, resulting in three new, as yet unassigned STs (S1 Table). Phylogenetic analysis of 215,629 concatenated SNPs identified among the core genome of the analyzed *E*. *coli* isolates revealed clustering consistent with the identified STs (Fig 1). Within MLST ST10, ST101, and ST58, the SNP analysis revealed higher discriminatory power than pure MLST. Isolates belonging to these STs were collected on different dates, from different dogs and possessed different resistance CDSs (Fig 1, S3 Table). No clustering according to the geographic origin was observed among the study isolates, as most isolates from KOS, AFG, UKR, and CRO revealed different MLST STs, CDSs contents, and phylogenetic SNP clustering (Fig 1 and S1 Table).

In three cases, identical clones were detected from different dogs living in close contact. First, the combination of *bla*_CTX-M-14_ and *bla*_TEM-1B_ was found in nine ST744 clonal isolates originating from five different dogs isolated within the same month (Fig 1 and S1 Table). Second, the six ST648 isolates, originating from two different dogs in the same month, were the only isolates found to contain the combination of *bla*_CTX-M-15_ and *bla*_OXA-1_ (Fig 1 and S1 Table). Finally, three ST410 isolates, collected from three different dogs over five months, were all found to contain *bla*_CTX-M-15_ and *bla*_OXA-1_. However, the isolate recovered five months after the other two did differ somewhat in that it was found to lack the *senB* gene (Fig 1 and Table 2). In one household, three dogs shed three ESBL-producing *E*. *coli* with identical MLST ST58 within 18 days, but these possessed SNP and gene content differences (GER_MD77_1507_Eco_075, GER_CD72_1507_082) (Fig 1 and S1 and S3 Tables). Focusing only on clonal isolates within longitudinal reshedding in individual dogs, a maximum isolation-time difference of seven days could be shown for five dogs (GER_MD03, GER_MD07, GER_MD08, GER_MD11, GER_MD14) (Fig 1 and S1 Table).

## Discussion

We collected 101 bacterial isolates during a 12-month ESBL-screening study of clinically healthy dogs and characterized their antimicrobial resistance phenotypes and genotypes through *in vitro* testing and whole genome sequence analysis. Here, 16 of 20 German military dogs (80%) that had no history of medical treatment for the previous twelve months were found to shed ESBL-producing bacteria at least once within the study period (S1 Table). Although a high prevalence of ESBL-producing bacteria is suspected in livestock, our findings were surprising considering that the investigated animals were clinically healthy and untreated [42]. Also concerning was the result that 9% of the characterized *E*. *coli* isolates from clinically healthy German dogs were completely resistant against all tested cephalosporins (COX, CTX, CAZ, and CEP) (S2 Table). As the microbiological resistance against third-generation cephalosporins in European countries was stated as generally low in a review of 2012 data provided by the European Centers for Disease Control (ECDC), this result can be interpreted as a trend towards increasing multidrug resistance [43].

The overall trends of increasing antimicrobial resistance have led to several actions in recent years. In 2015, increasing concern on the animal welfare consequences of antimicrobial resistance in bacteria from animal sources led to the establishment of a sub-committee for Veterinary Antimicrobial Susceptibility Testing (VetCAST) of the European Committee on Antimicrobial Susceptibility Testing (EUCAST) [44]. In 2018, the national German veterinary pharmacy regulation law was enforced. According to this law, if a veterinarian applies antibiotics to animals, the MIC of bacterial isolates must be determined in case of repeated or change of medication, rededication, or regarding therapy of flocks or regarding animals bred for specific purposes [45]. This enforcement was aimed at a reduction of the use of antibiotics, but as well as at avoiding an increase of antimicrobial resistance through non-suitable therapy. Since 2014, the amounts and application of antibiotics in animal husbandry in Germany are officially collected in a large database. The Federal Veterinary Surgeons' Association regularly publish guidelines for the prudent use of veterinary antimicrobial drugs, and may consider data regarding the use, but also antimicrobial resistance [46]. The data of the present study contribute to comprehend trends within the complex field of antimicrobial resistance.

Resistance against single antibiotics within the class of cephalosporins was common among the investigated isolates, with 14% of the evaluated isolates resistant to COX and 92% resistant to CEP (S2 Table). COX is a 2^nd^ generation cephamycin, frequently used in the treatment of dogs and other companion animals [46]. CEP is a 4^th^ generation cephalosporin limited to use in humans, making the 92% resistance rate observed here unexpectedly high (S2 Table) [47]. These results should be considered when revising the drug application recommendations for human and animal patients [21].

Among the currently available beta-lactams, the carbapenems, such as ERT and MER, are antibiotics of last resort. They are unique in that they are resistant to a high degree against hydrolysis by most beta-lactamases. They can sometimes act as “slow substrates” or inhibitors of beta-lactamases, and, yet, still target penicillin-binding proteins [2]. Although carbapenems are limited to use in humans only, off-label use or prescription may allow animals to be treated with these antibiotics [48]. In this study, we did not find any carbapenem resistance using the *in vitro* microbouillon dilution method (S2 Table). The *in silico* analysis detected similarly low levels of resistance, identifying only four isolates with a single carbapenemase CDS each (S4 Table). This suggests that dogs do not represent a likely source for the high rates of carbapenem resistance that have been published for hospital-acquired strains [49].

### Antimicrobial resistance–*in vitro* and *in silico* analyses

Initial isolate selection was based on growth on supposedly ESBL- selective Brilliance ESBL AGAR (Oxoid, Wesel, Germany) containing an unknown antibiotic-mix. The *in vitro* analysis and subsequent interpretation according to current CLSI guidelines revealed 88 out of 95 isolates to be actual ESBL-producers [21–22]. As there were no interpretation guidelines available for six of the investigated bacterial isolates, including the *Enterobacter* spp., the *Aeromonas spp*., and *Pseudomonas* species other than *P*. *aeruginosa* isolates, we did not assign these as ESBL-producers in S2 Table [21–22]. However, for one out of these isolates (*Aeromonas hydrophila)*, ESBL-activity according to the rule “more than a three twofold concentration decrease comparing growth in the presence of CTX and CTX in combination with clavulanic acid” was observed (S2 Table) [21]. Pure antimicrobial resistance without ESBL-activity revealed five out of the investigated isolates. One more isolate, GER_EN01_1501_Eco_087, revealed an intermediate status, and another isolate, GER_MD01_1509_Eco_059, did not even reveal antimicrobial resistance in the *in vitro* testing. Finally, the *Pseudomonas aeruginosa* isolate, GER_MD14_1510_Pae_083, did not reveal ESBL-activity, it was considered as a susceptible isolate according to the CLSI guidelines for CAZ and CEP (S2 Table) [21]. These results indicate some lack of specificity for the selective Brilliance ESBL AGAR (Oxoid, Wesel, Germany). We compared the *in vitro* results with the detection of ESBL-specific CDSs in the *in silico* analysis (S5 Table). The susceptible isolate GER_MD01_1509_Eco_059, and the intermediate isolate GER_EN01_1501_Eco_087 revealed a single ESBL-CDS each, *bla*_TEM-1B_ and *bla*_OXA-1_, respectively (S4 Table). In contrast, the two *in vitro* antimicrobial resistant isolates GER_EN02_1501_Eco_088 and GER_MD10_1505_Pmi_049 did not reveal any ESBL-CDSs at all (S5 Table). Although the results from the two methodologies do not match entirely, we consider this a fairly high level of concordance between the *in vitro* and *in silico* analyses. It further suggests that the initial screening method for ESBL-producers was not highly specific, as eight isolates could grow on the selective media, but did not reveal true ESBL-properties (S2 Table).

The isolates GER_MD03_1507_Eco_050 and GER_MD11_1505_Eco_023 were found to possess only a single beta-lactamase gene (S4 Table). However, they revealed multiple resistance *in vitro*, against CTX and CEP, and were identified as ESBL-producers (S2 Table). *In vitro* and *in silico* correlation is therefore still too complex to predict a particular resistance from the result of a single detected beta-lactamase gene. Nevertheless, amongst the *E*. *coli* ESBL-producers, the most frequent ESBL-specific CDSs were *bla*_CTX-M1_, *bla*_CTX-M15_, *bla*_TEM-1B_, and *bla*_OXA-1_ in the present study, as it has been published (Table 1) [3, 11].

The investigated isolates belonged to six different bacterial species. Noticeably, the *in silico* results showed strict bacterial species-specific CDS patterns regarding antimicrobial resistance (Table 1). Although species specificity has been described for some of these resistance genes such as the “*Pseudomonas aeruginosa* cephalosporinase” (*bla*_PAO_) [50], other classes can be found within various bacterial species belonging to the family Enterobacteriaceae such as the “sulphydryl variable class A beta-lactamase” (*bla*_SHV_) [1]. Finally, some beta-lactamases, such as the “oxacillin-hydrolyzing beta-lactamase” (*bla*_OXA_), and the “cefotaximase-Munich extended-spectrum beta-lactamase” (*bla*_CTX-M_), were described for genetically distant bacterial genera such as the Gram-positive *Enterococcus* and Gram-negative *Escherichia* [51]. Apart from their presence, enzymes may also vary regarding their kinetic activity. The “Temoneira class A beta-lactamases” (TEM-1) are able to hydrolyze ampicillin at a greater rate than carbenicillin, oxacillin, or cephalothin, and have negligible activity against extended-spectrum cephalosporins. Similar findings have been published for the CTX-M and OXA beta-lactamase subtypes [1]. The isolate GER_MD90_1604_Eco_099 was revealed to be resistant against 2^nd^ and 3^rd^ generation cephalosporins, COX, CTX, and CAZ (S2 Table). As previously published, a single resistance CDS, the cephamycinase *bla*_CMY-2_, was likely responsible for this phenotype. Interestingly, this isolate was not an ESBL-producer by definition (S4 Table) [14]. Therefore, neither of the methods, either the phenotypic characterization nor whole genome analysis, can completely replace the other due to lack of crucial information. Thus, it is currently not possible to predict the phenotype using pure whole genome analysis and vice versa. However, due to its relevance for clinical diagnostics and treatment recommendations, the *in vitro* analysis will likely remain the gold standard at this time [21]. Drawbacks to this method include the fact that inoculum effects and *in vitro* conditions may affect MIC measurements, which may obscure a true underlying resistance genotype in various bacterial species [51–54]. In addition, non-Enterobacteriaceae organisms are currently not considered in CLSI guidelines for ESBL detection, impeding treatment recommendations for clinical patients affected by other species [1, 21].

In previous studies, PCR detection was used to identify individual CDSs of beta-lactamase subgroups of *bla*_CTX-M_, *bla*_CMY_, *bla*_TEM_, *bla*_SHV_, *bla*_PSE_, *bla*_OXA_, *bla*_AmpC_, *bla*_ACC_ in isolates originating from companion animals [3, 4, 10–12, 51, 55–60]. By utilizing whole genome sequencing, we were able to identify seven additional beta-lactamase types and subtypes, including, *bla*_SFO_, *bla*_Cph_, *bla*_VIM_, *bla*_Act_, *bla*_MIR_, *bla*_MOX_, and *bla*_PAO_ (Table 1 and S4 Table). Similar findings have been published after the analysis of human derived ESBL-producing bacteria, reflecting the superior detection capabilities offered by whole genome sequence analysis [17]. In summary, not using whole genome sequence analysis, an investigator risks missing crucial information concerning antibiotic resistance that could be helpful and sometimes even crucial for subsequent epidemiological interpretation.

### Prevalence of specific MLST STs and resistance genes

By the year 2000, a CTX-M beta-lactamase producing ST131 *E*. *coli* was recognized as a clone with worldwide prevalence, with about half of all hospital acquired ESBL-infections associated with this sequence type [17, 61–63]. In the present study, ST131 was not detected among the dog-derived isolates, suggesting that this ST might be less adapted to the canine host [64]. The two most common MLST ST identified in the present study were ST744 and ST10, with nine and eight isolates among the 85 isolates, respectively (Fig 1 and S1 Table). Several predominant ESBL-producing *E*. *coli* lineages have been identified for animals. The MLST ST10 was repeatedly isolated from pigs in Ireland, ST410 from small animals in Switzerland, and finally ST38 and ST131 from poultry and small animals in the Netherlands [51, 65–67]. As the MLST ST10 dog-isolates of the present study originated from Germany, Kosovo, Ukraine and Afghanistan, a significant geographic cumulation of MLST ST10 cannot be concluded from the present data (Fig 1 and S1 Table).

Regarding the transmission and prevalence of certain beta-lactamase subtypes, it has been suggested that human isolates hosting the CTX-M beta-lactamase subtypes vary by geographic origin [17]. In Germany, the plasmid coded *bla*_CTX-M-15_ gene is the most frequent subtype originating from human patient isolates [7, 62]. In the present study, *bla*_CTX-M-15_ was detected in 30% (28 isolates with the gene out of 89 true ESBL-producers) of the isolates originating from Germany, however also from the countries Kosovo, Ukraine, Croatia, and Afghanistan (S4 Table). As an additional 54% of isolates were found to carry *bla*_CTX-M_-subtypes other than *bla*_CTX-M-15_ (S4 Table), it may be assumed that the CTX-M beta-lactamases have a generally high prevalence, regardless of source.

A recent publication indicated large-scale transmission of hospital-associated *bla*_IMP_-carrying isolates into wildlife after feeding of birds at a local waste depot [6]. But this finding could not be supported by results of a study from the same year with hardly any confirmed transmission from 22 ESBL-positive humans to their companion dogs [68]. Hypotheses regarding transmission pathways and reservoirs are often oversimplified in single studies whereas the reality is far more complex [42].

### Phylogeny

The bacterial species *E*. *coli* possesses great genetic diversity, with >7,000 identified MLST STs [27]. Although the vast majority of *E*. *coli* is a prolific commensal part of the gut microbiome, selected serotypes cause serious disease, including the enterohemorrhagic or extraintestinal pathogenic *E*. *coli* (EHEC, ExPEC), which express various virulence and toxin genes [64, 69–70]. Outbreak investigation revealed that an epidemiological linkage was estimated if two isolates revealed the same MLST ST, and differed by less than ten core SNPs [17, 71]. In contrast, antimicrobial resistance in *E*. *coli* is not restricted to specific clones, as it has been identified in a broad variety of genotypes isolated from human and animal sources [11, 42, 51, 67, 70, 72]. We observed representative diversity among the 85 ESBL-producing *E*. *coli* characterized here, with 34 different MLST STs, including three currently unassigned STs (Fig 1 and S1 Table). Amongst the isolates belonging to MLST ST10, additional SNP diversity could be identified, likely related to the different countries of origin for these isolates (Fig 1 and S3 Table).

In the present study, four clusters (ST744, ST648, ST410, ST23) were identified that included isolates of different dog-origin that also showed similar beta-lactamase CDS profiles and lacked SNP differences (Fig 1). This suggests that these isolates epidemiologically share the same ancestor, which may be explained by mutual/ reciprocal transmission, as the dogs in question regularly share the same training facility and runout (Fig 1). Similar findings were recently published where low genetic diversity was described for 297 ST131 *E*. *coli* strains isolated in a longitudinal study from a group of patients living in a long-term care facility, indicating acquisition from a common source or person-to-person transmission [63].

## Supporting information

S1 Table Origin of the studied isolates, MLST ST data and virulence CDSs. (XLSX)

S2 Table Results of the microbouillon dilution method and interpretation of the MICs according to CLSI guidelines in green (susceptible), yellow (intermediate) and red (resistant) [21–22]. (XLSX)

S3 Table GenBank references for the genomes analysed in the present study. (XLSX)

S4 Table Results of the *in silico* analysis regarding antimicrobial resistance CDSs with the large-scale blast score ratio (LS-BSR) pipeline. (XLSX)

S5 Table Direct comparison of the *in vitro* and *in silico* data. (XLSX)
